# Back-exchange of deuterium in neutron crystallography: characterization by IR spectroscopy

**DOI:** 10.1107/S1600576717003624

**Published:** 2017-03-24

**Authors:** Ai Woon Yee, Matthew P. Blakeley, Martine Moulin, Michael Haertlein, Edward Mitchell, V. Trevor Forsyth

**Affiliations:** a Life Sciences Group, Institut Laue–Langevin, 71 avenue des Martyrs, Grenoble 38042, France; b Faculty of Natural Sciences, Keele University, Keele ST5 5BG, UK; c Large-Scale Structures Group, Institut Laue–Langevin, 71 avenue des Martyrs, Grenoble 38042, France; d European Synchrotron Research Facility, 71 avenue des Martyrs, Grenoble 38043, France

**Keywords:** D_2_O/H_2_O back-exchange, neutron crystallography, IR spectroscopy, deuteration

## Abstract

The application of IR spectroscopy to the characterization and quality control of samples used in neutron crystallography is described. While neutron crystallography is a growing field, the limited availability of neutron beamtime means that there may be a delay between crystallogenesis and data collection. Since essentially all neutron crystallographic work is carried out using D_2_O-based solvent buffers, a particular concern for these experiments is the possibility of H_2_O back-exchange across reservoir or capillary sealants. This may limit the quality of neutron scattering length density maps and of the associated analysis. Given the expense of central facility beamtime and the effort that goes into the production of suitably sized (usually perdeuterated) crystals, a systematic method of exploiting IR spectroscopy for the analysis of back-exchange phenomena in the reservoirs used for crystal growth is valuable. Examples are given in which the characterization of D_2_O/H_2_O back-exchange in transthyretin crystals is described.

## Abstract 2

The application of IR spectroscopy to the analysis of D_2_O/H_2_O back-exchange in samples used for neutron crystallography is described.

## 1. Introduction   

The use of neutron crystallography to study biological macromolecules has been expanding in recent years (Blakeley *et al.*, 2015 ▸). The technique is of particular importance for the study of protonation states, protein–water interactions and protein–ligand interactions. It allows the details of intricate hydrogen-bonding networks to be probed, as well as the observation of more exotic ionic species (*e.g.* H_3_O^+^). Specific examples include the study of enzyme mechanisms (Casadei *et al.*, 2014 ▸; Oksanen *et al.*, 2014 ▸; Kwon *et al.*, 2016 ▸), protein–drug interactions (Weber *et al.*, 2013 ▸; Gerlits *et al.*, 2016 ▸) and redox systems (Cuypers *et al.*, 2013 ▸). The advantage of using neutrons is that the coherent scattering lengths for protium (^1^H) and deuterium (^2^H) are of a similar magnitude to those of other common elements of a macromolecule (*e.g.* C, N, O and S). This means that, in contrast with the situation for X-ray diffraction, they have clear visibility in neutron crystallographic studies. However, there are several factors to be considered when studying biological macromolecules with neutrons. The neutron coherent scattering length of protium carries a negative sign, which can cause cancellation effects in neutron scattering length density maps (hereafter referred to as neutron maps) (Fisher *et al.*, 2014 ▸). Another important point is that protium, which accounts for about half of the atoms in biological macromolecules, has a very large in­coherent scattering cross section (80.27 barn; 1 barn = 100 fm^2^), arising from the two spin states of the atom. This incoherent scattering severely impairs the accuracy with which the coherent crystallographic data can be measured. This problem can be largely avoided by replacing protium with its isotope, deuterium, which has a much lower incoherent scattering cross section (2.05 barn) and a positive coherent scattering length.

Neutron macromolecular crystallography therefore benefits from the use of deuteration – ideally perdeuteration, where all protein and solvent protium atoms are replaced by deuterium. Perdeuterated protein is typically produced *in vivo* and purified using protiated reagents and solvents (Haertlein *et al.*, 2016 ▸). In the final stages of purification and crystallization, the H_2_O solvent is usually replaced by D_2_O. In addition to replacing the solvent (∼40–50% of the crystal volume), this, in principle, also results in the replacement of solvent-accessible labile protium atoms (∼20–25%) on the protein backbone and side-chains (*i.e.* N—H, S—H and O—H groups). The routine use of perdeuterated crystals has been one of the major developments for neutron macromolecular crystallography in recent years (Blakeley *et al.*, 2008 ▸; Gardberg *et al.*, 2010 ▸; Tomanicek *et al.*, 2011 ▸; Howard *et al.*, 2011 ▸, 2016 ▸; Munshi *et al.*, 2012 ▸; Cuypers *et al.*, 2013 ▸; Haupt *et al.*, 2014 ▸; Laulumaa *et al.*, 2015 ▸). It reduces the crystal volume needed for data collection by at least an order of magnitude compared with D_2_O-soaked/grown protiated crystals. However, despite recent developments (Blakeley *et al.*, 2015 ▸), the crystal volumes typically required are still ∼0.10 mm^3^, several orders of magnitude larger than those normally used for X-ray crystallography. Given the high demand for neutron beamtime, and the time and effort required for crystallogenesis and data collection, it is of crucial importance to ensure that the samples produced are of the best quality and have the highest possible exchange of protium by deuterium. There is currently no method that is used systematically to assess the D_2_O/H_2_O content of crystals prior to data collection. This is a major difficulty, given that back-exchange of D_2_O by H_2_O can occur at various stages during crystallogenesis and/or sample mounting (Bouquiere *et al.*, 1993 ▸, 1994 ▸), and this may impact on data quality and complicate subsequent interpretation.

**Figure 1:**
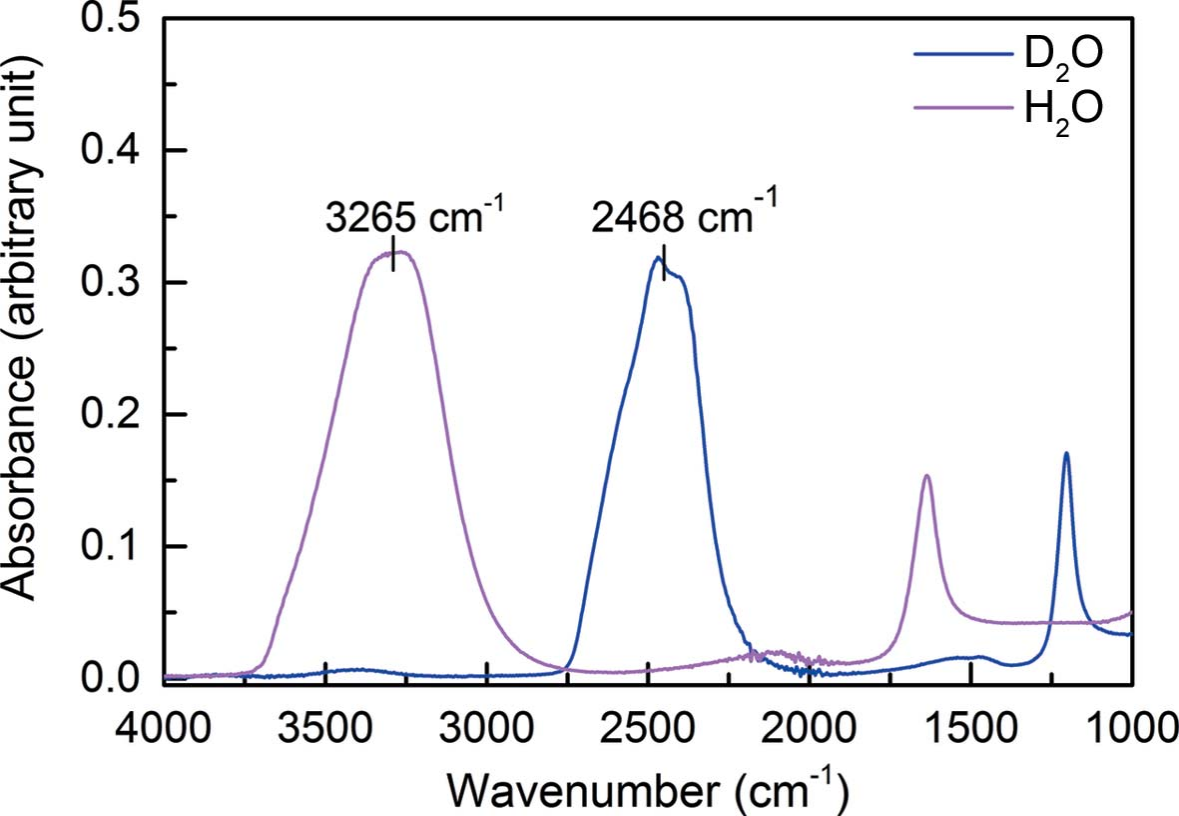
FT–IR absorption spectra of liquid H_2_O (magenta) and liquid D_2_O (blue) measured at room temperature. The resonance frequency of the O—H stretch of H_2_O is at 3265 cm^−1^, whereas that of the O—D stretch of D_2_O is at 2468 cm^−1^.

Here we describe a method using Fourier transform IR (FT–IR) spectroscopy to monitor the composition of protein solvent prior to and after crystallization, and after sample mounting. Fig. 1 ▸ shows the absorption spectra of pure liquid H_2_O and liquid D_2_O at room temperature. H_2_O has a characteristic resonance frequency at ∼3265 cm^−1^ because of the existence of the O—H functional group, whereas D_2_O resonates at a lower frequency of ∼2468 cm^−1^ owing to the stronger O—D bond (Scheiner & Čuma, 1996 ▸). Since the main interest here is to monitor the ratio of H_2_O/D_2_O in the sample, we focus on the ratio of these two peaks and its significance for exchange in solvent and protein side-chain groups. This of course also has implications for the exchange of labile protium atoms on the backbone amide groups. For example, at the data collection stage it is possible to record FT–IR data from the mother liquor held in the sample capillary. A fundamental (but reasonable) assumption is that a spectrum recorded in this way will reflect both the solvent content in the crystal and the deuterium exchange in the protein.

## 2. Methods   

### 2.1. Sample preparation and crystallization   

The protein samples used were deuterated recombinant transthyretin (TTR) protein. The details of the expression and purification have been described previously (Haupt *et al.*, 2011 ▸; Yee *et al.*, 2016 ▸). While the deuterated TTR was expressed under conditions where only deuterium atoms were present, protiated solutions were used during purification and it was assumed that the labile deuterium atoms in the protein were exchanged by protium. Prior to protein crystallization, the purified protein was buffer-exchanged using a deuterated solution so that the labile protium atoms would be replaced by deuterium. Deuterated TTR crystals were then grown at 291 K using sitting-drop vapour diffusion in deuterated malonate buffer [sodium malonate was dissolved in D_2_O, and the pD was adjusted using malonic acid which had been dehydrated (by heat and vacuum) and redissolved in D_2_O (repeated three times)]. Crystal *A* was crystallized in 2.1 *M* malonate pD 6.4 in a 1:1 protein-to-buffer ratio and a protein concentration of 20 mg ml^−1^, whereas crystal *B* was crystallized in 1.9 *M* malonate pD 6.4 with a 7:5 protein-to-buffer ratio and a protein concentration of 25 mg ml^−1^. Both crystals were grown to ∼0.5 mm^3^ using the same TTR protein variant and were of space group *P*2_1_2_1_2 with unit-cell dimensions of *a* = 43.8 Å, *b* = 86.3 Å and *c* = 65.5 Å. For neutron data collection, the crystals were mounted in quartz capillaries and surrounded by a small amount of mother liquor from the crystallization well. The capillary was sealed tightly using wax to eliminate the diffusion of gas and atmospheric water.

### 2.2. X-ray and neutron data collection and processing   

Details of the neutron and X-ray data collection approaches that have been used previously for TTR have been reported by Haupt *et al.* (2014 ▸). Neutron quasi-Laue diffraction data were collected at room temperature using the LADI-III instrument at the ILL (Blakeley *et al.*, 2010 ▸). Data were processed to a maximum resolution of 1.8 Å. X-ray diffraction data were recorded on the beamlines MASSIF-1 (Bowler *et al.*, 2015 ▸) and ID30B at the ESRF (Mueller-Dieckmann *et al.*, 2015 ▸) for crystals *A* and *B*, respectively. Data were recorded at room temperature on the same capillary-mounted crystals as used for data collection on LADI-III. Data were processed to the same maximum resolution of the corresponding neutron data, *i.e.* 1.8 Å. PDB code 5clx was used as the starting model for joint X-ray and neutron refinement. The neutron *R*_work_ and *R*_free_ values for the final model of crystal *A* were 0.202 and 0.238, respectively, while the X-ray *R*_work_ and *R*_free_ values were 0.173 and 0.205, respectively. For crystal *B*, the *R*_work_ and *R*_free_ values were 0.211 and 0.260 for neutrons, and 0.172 and 0.210 for X-rays, respectively.

### 2.3. FT–IR spectroscopy measurement   

The FT–IR spectrometer used was an FT/IR-4600 (JASCO). It is equipped with an ATR Pro One sample cell, a single-reflection ATR with a monolithic diamond crystal, which can be used for the analysis of aqueous or solid samples. For the measurement of a solid sample, a compression clamp is used to ensure that the sample is in contact with the ATR crystal. A background spectrum was first obtained from an empty sample cell. 1.5 µl of liquid sample was then pipetted into to the sample cell for measurement. Liquid samples can be pipetted from the mother liquor surrounding the crystal in the capillary or directly from the crystallization plate, or from any buffer solution used during purification and crystallization of protein. The current design of the machine does not allow measurements to be performed directly on the sample in the capillary because of the compression clamp. Sample spectra (16 scans) were recorded over a wavenumber range of 4000–400 cm^−1^ (resolution 4 cm^−1^). The background data were subtracted from the sample spectra.

## 3. Results   

**Figure 2:**
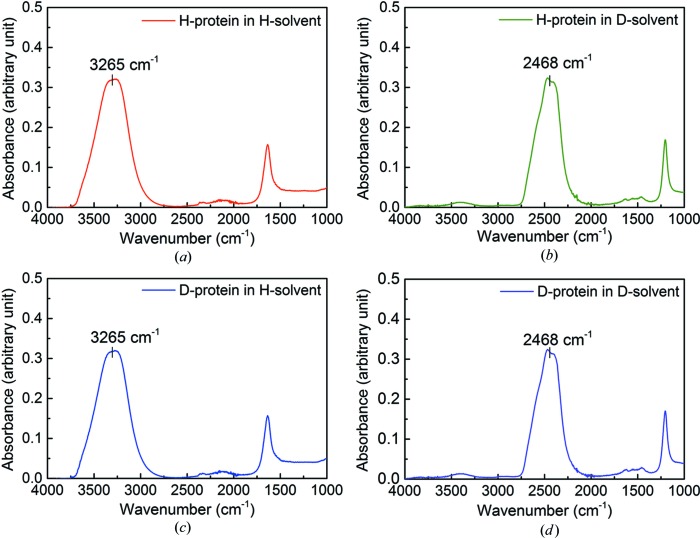
FT–IR absorption spectra of protiated TTR protein in (*a*) protiated solvent and (*b*) deuterated solvent; and of deuterated TTR protein in (*c*) protiated solvent and (*d*) deuterated solvent. The characteristic O—H and O—D peaks are seen at 3265 and 2468 cm^−1^, respectively.

The aim of the FT–IR measurements was to provide information regarding the ratio of protium and deuterium atoms present in samples used for neutron crystallographic experiments. This method probes the solvent and labile hydrogen-atom content of the sample and is not affected by the non-exchangeable protium/deuterium atoms of the protein itself. Hence, as shown in Fig. 2 ▸, both deuterated and protiated protein show essentially identical spectra (Figs. 2 ▸ *a* and 2 ▸ *c*) when placed in the same protiated solvent, with a dominant peak at 3265 cm^−1^. Similarly, the spectra corresponding to protiated protein and deuterated protein (Figs. 2 ▸ *b* and 2 ▸ *d*) in deuterated buffer show no significant differences, with a peak at 2468 cm^−1^.

This method can therefore be applied to analyse solvent exchange at various stages from purification through to crystallization. The solvent environment in the crystallization well is assumed to be in equilibrium with the solvent environment of the crystal. Hence, by analysing the solvent composition of the well using FT–IR it is possible to infer the solvent composition of the crystal.

### 3.1. Back-exchange of solvent molecules   

**Figure 3:**
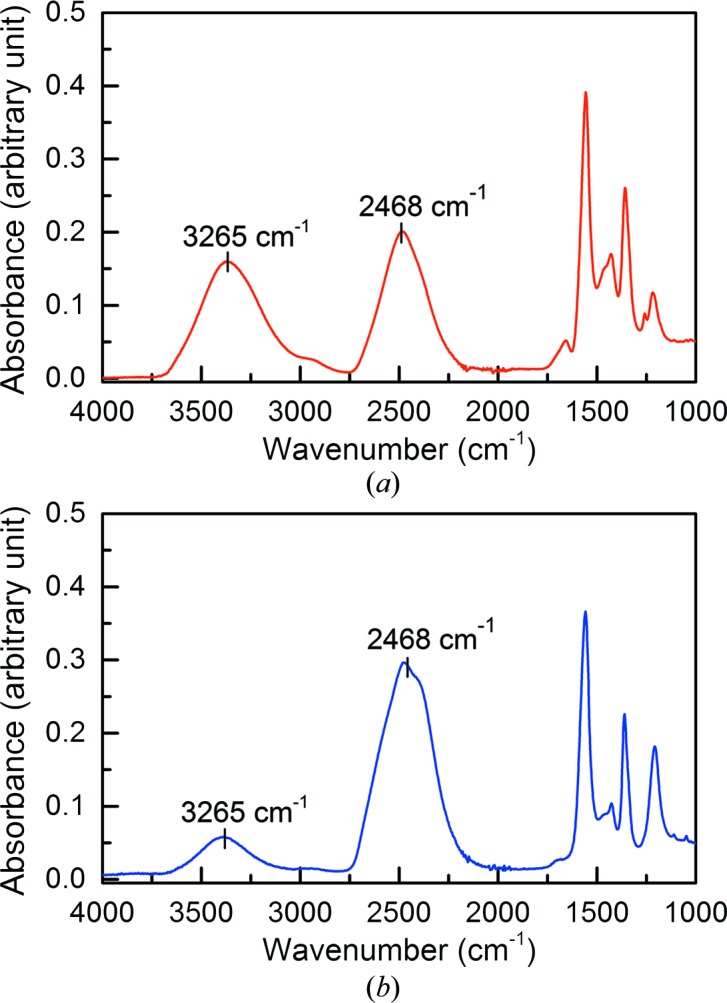
FT–IR absorption spectra of (*a*) the capillary solvent of a deuterated TTR crystal that has suffered severe back-exchange (crystal *A*) and (*b*) that of a crystal of the same variant that had only a little back-exchange (crystal *B*).

The Matthew’s coefficient calculation (*V*_m_ = 2.09 Å^3^ Da^−1^) suggests that the solvent content of the crystals is ∼41.2% (both crystals *A* and *B* were produced from the same TTR variant and crystallized in the same space group and unit cell). Fig. 3 ▸ shows the FT–IR absorption spectrum of the capillary solvent of a deuterated TTR crystal that has suffered severe back-exchange (crystal *A*) and that of a crystal of the same variant that has undergone only mild back-exchange (crystal *B*). The estimated percentages of H_2_O (absorbance at 3265 cm^−1^) and D_2_O (absorbance at 2468 cm^−1^) are shown in Table 1 ▸. About 40% of the solvent content in crystal *A* has been back-exchanged to H_2_O. This mode of exchange may occur for a number of reasons, including permeability of the sealing films used in crystallization wells or of the porous wax used as a capillary sealant, or following exposure to the environment during transfer of the crystal from the well to the capillary.

**Figure 4:**
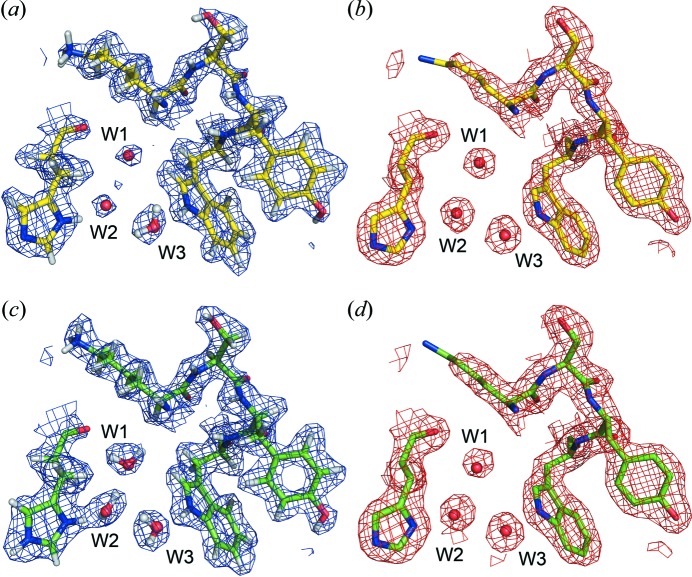
Part of (*a*) the 2*F*_o_–*F*_c_ neutron map (contoured at 1.5 σ) and (*b*) the 2*F*_o_–*F*_c_ X-ray map (contoured at 1.5σ) of chain *A* of crystal *A*. The neutron scattering length density for the three ordered water molecules (W1, W2 and W3) is compromised, indicating the back-exchange of D_2_O. (*c*) The 2*F*_o_–*F*_c_ neutron map (contoured at 1.5σ) and (*d*) the 2*F*_o_–*F*_c_ X-ray map (contoured at 1.5σ) of crystal *B*, corresponding to the same region shown in panels (*a*) and (*b*). The neutron scattering length density for the three water molecules can be clearly seen.

Figs. 4 ▸(*a*) and 4 ▸(*c*) show 2*F*_o_–*F*_c_ neutron maps of the same region in crystals *A* and *B*, respectively (both to 1.8 Å resolution). Three ordered water molecules are known to be present in this part of the molecule. When both maps are contoured at 1.5σ, the neutron map of crystal *B* (Fig. 4 ▸ *c*), where the percentage of H_2_O content is only 10% (Table 1 ▸), identifies the position and orientation of these three water molecules clearly. In contrast, cancellation effects in the corresponding neutron map for crystal *A* (Fig. 4 ▸ *a*) have compromised the ability to model deuterium atoms effectively for the water molecules. The high level of H_2_O absorbance measured with FT–IR correlates with the lack of visibility for the deuterium atoms of these ordered water molecules in crystal *A*, confirming the global level of back-exchange. As shown in Figs. 4 ▸(*b*) and 4 ▸(*d*), the X-ray electron density maps for crystals *A* and *B* are identical, as D_2_O and H_2_O are not distinguishable by X-ray crystallography.

## 4. Discussion and conclusion   

This paper demonstrates that FT–IR spectroscopy can be used in a routine way to assess back-exchange during sample preparation and also both before and after data collection. It is highly desirable to avoid back-exchange that could occur during data collection, since the final dataset may then contain a mixture of exchanged populations. It is perfectly feasible to conceive of an *in situ* FT–IR system on neutron diffractometers that would allow back-exchange to be monitored during data acquisition, and this could help to optimize the use of beamtime. Complementary spectroscopic arrangements of this type are widely used on X-ray crystallography beamlines (von Stetten *et al.*, 2015 ▸).

While it is clear that solvent back-exchange occurs almost uniformly throughout the protein, that of the amide groups of the protein backbone is highly dependent on the region of the protein structure. It is perhaps not surprising that different regions of a biological macromolecule may exchange solvent deuterium/protium atoms at different rates, depending on accessibility and protein stability (Bennett *et al.*, 2008 ▸). Analogous approaches have been pursued for H/D exchange studies using mass spectrometry (Konermann *et al.*, 2011 ▸; Wales & Engen, 2006 ▸) and have provided powerful insights to protein flexibility (Mehmood *et al.*, 2012 ▸; Giladi *et al.*, 2016 ▸). It is possible that neutron crystallography could exploit this type of methodology in the future.

## Figures and Tables

**Table 1:** FT–IR absorbance values at 3265 and 2468 cm^−1^ and the corresponding estimated percentages of H_2_O and D_2_O content in the capillary solvent of crystals *A* and *B*

	Crystal *A*	Crystal *B*
Absorbance at 3265 cm^−1^ (arbitrary units)	0.1307	0.0320
Absorbance at 2468 cm^−1^ (arbitrary units)	0.1976	0.2897
H_2_O content (%)	39.8	9.9
D_2_O content (%)	60.2	90.1
